# Supplementary material for: Experience-dependent weakening of callosal synaptic connections in the absence of postsynaptic FMRP
Source: eLife. 2021 Oct 7;10:e71555. doi: 10.7554/eLife.71555 (PMC8526058; doi:10.7554/eLife.71555)
Supplement: Transparent reporting form [file elife-71555-transrepform1.docx]

***eLife’s* transparent reporting form**

We encourage authors to provide detailed information *within their submission* to facilitate the interpretation and replication of experiments. Authors can upload supporting documentation to indicate the use of appropriate reporting guidelines for health-related research (see [EQUATOR Network](http://www.equator-network.org/%20)), life science research (see the [BioSharing Information Resource](https://biosharing.org/)), or the [ARRIVE guidelines](http://www.plosbiology.org/article/info:doi/10.1371/journal.pbio.1000412) for reporting work involving animal research. Where applicable, authors should refer to any relevant reporting standards documents in this form.

If you have any questions, please consult our Journal Policies and/or contact us: [editorial@elifesciences.org](mailto:editorial@elifesciences.org).

**Sample-size estimation**

- You should state whether an appropriate sample size was computed when the study was being designed
- You should state the statistical method of sample size computation and any required assumptions
- If no explicit power analysis was used, you should describe how you decided what sample (replicate) size (number) to use

Please outline where this information can be found within the submission (e.g., sections or figure legends), or explain why this information doesn’t apply to your submission:

We didn’t perform sample size calculation prior to the experiments. However, all datasets in this study have a sample size similar to other published studies in related fields.

**Replicates**

- You should report how often each experiment was performed
- You should include a definition of biological versus technical replication
- The data obtained should be provided and sufficient information should be provided to indicate the number of independent biological and/or technical replicates
- If you encountered any outliers, you should describe how these were handled
- Criteria for exclusion/inclusion of data should be clearly stated
- High-throughput sequence data should be uploaded before submission, with a private link for reviewers provided (these are available from both GEO and ArrayExpress)

Please outline where this information can be found within the submission (e.g., sections or figure legends), or explain why this information doesn’t apply to your submission:

Biological replicates are the number of the cell (pairs) recorded and this is indicated in the Methods and figure legends. An outlier was encountered in Figure 5 – figure supplement 2 (lower left panel) and was excluded based on statistical test. Exclusion/inclusion of individual cell recordings was based on cell health/quality parameters, which is described in Methods under Electrophysiology section.

**Statistical reporting**

- Statistical analysis methods should be described and justified
- Raw data should be presented in figures whenever informative to do so (typically when N per group is less than 10)
- For each experiment, you should identify the statistical tests used, exact values of N, definitions of center, methods of multiple test correction, and dispersion and precision measures (e.g., mean, median, SD, SEM, confidence intervals; and, for the major substantive results, a measure of effect size (e.g., Pearson's r, Cohen's d)
- Report exact p-values wherever possible alongside the summary statistics and 95% confidence intervals. These should be reported for all key questions and not only when the p-value is less than 0.05.

Please outline where this information can be found within the submission (e.g., sections or figure legends), or explain why this information doesn’t apply to your submission:

Statistical analysis process is described in the Methods. Also, statistical tests used for individual experiments, summary statistics and p-values were reported in the figure legend and occasionally in the main text.

(For large datasets, or papers with a very large number of statistical tests, you may upload a single table file with tests, Ns, etc., with reference to sections in the manuscript.)

**Group allocation**

- Indicate how samples were allocated into experimental groups (in the case of clinical studies, please specify allocation to treatment method); if randomization was used, please also state if restricted randomization was applied
- Indicate if masking was used during group allocation, data collection and/or data analysis

Please outline where this information can be found within the submission (e.g., sections or figure legends), or explain why this information doesn’t apply to your submission:

Allocation of mice or neurons into experimental groups was based on the genotype of the mice and/or the fluorescence of the neurons. No blinding was performed during data acquisition. Especially for dual patch clamp recordings, knowing the identity of neurons by presence or absence of fluorescence is necessary for the researcher. Researcher practiced self-blinding during data analysis by hiding the identity of neurons until the whole dataset had been analyzed.

**Additional data files (“source data”)**

- We encourage you to upload relevant additional data files, such as numerical data that are represented as a graph in a figure, or as a summary table
- Where provided, these should be in the most useful format, and they can be uploaded as “Source data” files linked to a main figure or table
- Include model definition files including the full list of parameters used
- Include code used for data analysis (e.g., R, MatLab)
- Avoid stating that data files are “available upon request”

Please indicate the figures or tables for which source data files have been provided:

An Excel file containing the numerical data represented as a graph has been uploaded for each figure/supplement figure as ‘Source data’. Custom data collection and analysis software written based on Labview will be made available on Github or through direct communication to the corresponding authors.
